# The Functional Impact of Alternative Splicing on the Survival Prognosis of Triple-Negative Breast Cancer

**DOI:** 10.3389/fgene.2020.604262

**Published:** 2021-01-14

**Authors:** Sijia Wu, Jiachen Wang, Xinchao Zhu, Jacqueline Chyr, Xiaobo Zhou, Xiaoming Wu, Liyu Huang

**Affiliations:** ^1^School of Life Sciences and Technology, Xidian University, Xi’an, China; ^2^Center for Computational Systems Medicine, School of Biomedical Informatics, The University of Texas Health Science Center at Houston, Houston, TX, United States; ^3^Key Laboratory of Biomedical Information Engineering of Ministry of Education, School of Life Sciences and Technology, Xi’an Jiaotong University, Xi’an, China

**Keywords:** alternative splicing, triple-negative breast cancer, recurrence-free survival, overall survival, SRPK3, ITGB3, PCYT2

## Abstract

**Purpose:**

Triple-negative breast cancer (TNBC) is a type of breast cancer (BC) showing a high recurrence ratio and a low survival probability, which requires novel actionable molecular targets. The involvement of alternative splicing (AS) in TNBC promoted us to study the potential roles of AS events in the survival prognosis of TNBC patients.

**Methods:**

A total of 150 TNBC patients from The Cancer Genome Atlas (TCGA) were involved in this work. To study the effects of AS in the recurrence-free survival (RFS) prognosis of TNBC, we performed the analyses as follows. First, univariate Cox regression model was applied to identify RFS-related AS events. Their host genes were analyzed by Metascape to discover the potential functions and involved pathways. Next, least absolute shrinkage and selection operator (LASSO) method was used to select the most informative RFS-related AS events to constitute an AS risk factor for RFS prognosis, which was evaluated by Kaplan–Meier (KM) and receiver operating characteristic (ROC) curves in all the data and also in different clinical subgroups. Furthermore, we analyzed the relationships between splicing factors (SFs) and these RFS-related AS events to seek the possibility that SFs regulated AS events to influence RFS. Then, we evaluated the potential of these RFS-related AS events in the overall survival (OS) prognosis from all the above aspects.

**Results:**

We identified a total of 546 RFS-related AS events, which were enriched in some splicing and TNBC-associated pathways. Among them, seven RFS-related events were integrated into a risk factor, exhibiting satisfactory RFS prognosis alone and even better performance when combined with clinical tumor–node–metastasis stages. Furthermore, the correlation analysis between SFs and the seven AS events revealed the hypotheses that SRPK3 might upregulate PCYT2_44231_AA to have an effect on RFS prognosis and that three other SFs may work together to downregulate FLAD1_7874_RI to influence RFS prognosis. In addition, the seven RFS-related AS events were validated to be promising in the OS prognosis of TNBC as well.

**Conclusion:**

The abnormal AS events regulated by SFs may act as a kind of biomarker for the survival prognosis of TNBC.

## Introduction

Alternative splicing (AS) contributes to transcriptional diversity and plasticity by selecting which transcript isoforms are produced in a specific cell at a given time (Park et al., 2018). In humans, about 95% of genes with multiple exons are alternatively spliced to give rise to different protein isoforms (Kim et al., 2018). Besides being a critical mechanism during cell development, cell differentiation, and regulation of cell-type-specific functions, aberrant AS is also involved in multiple diseases, including triple-negative breast cancer (TNBC) (Chan et al., 2017; Climente-González et al., 2017).

Triple-negative breast cancer (BC), a kind of BC that does not express the genes for estrogen receptor, progesterone receptor, and HER2/neu (Foulkes et al., 2010), is considered to be more aggressive, have a poorer prognosis, and show a higher recurrence ratio than other types of BCs (Zhu et al., 2016). Currently, chemotherapy remains the standard therapeutic approach for TNBC at all stages. The lack of targeted therapies, high recurrence ratio, and low survival probability have fostered a major effort to discover actionable molecular targets which could effectively differentiate between TNBC patients with better survival and others (Bianchini et al., 2016).

Some previous studies about AS might provide insights into TNBC survival prognosis, which identified the dysregulation of splicing as a pathological factor of TNBC. For example, the widespread intron retention caused by splicing modulator E7107 is involved in multiple basal-like TNBC dependencies, including protein homeostasis, mitosis, and apoptosis (Chan et al., 2017), AS of CPEB2 drives anoikis resistance and the metastasis of TNBC (Johnson et al., 2015), TDP43 alters most splicing events with splicing factor (SF) SRSF3 in the regulation of TNBC progression (Ke et al., 2018), and the splicing regulations of PAR3 and NUMB exon 12 mediate the effect of reduced metastasis and proliferation of TNBC upon knockdown of TDP43 or SRSF3 (Ke et al., 2018). All these studies demonstrated the AS events to be potentially druggable and targetable molecular targets.

Thus, AS events deserved to be studied deeply in this work to discover their potential impacts on the survival of TNBC. To analyze the effects of AS events on the prognosis of recurrence-free survival (RFS), we first performed univariate risk factor analysis together with a regression model to identify the most dominant AS events with regard to RFS. Second, Kaplan–Meier (KM) curves and the receiver operating characteristic (ROC) curves were applied to test the prognosis ability of the risk factor composed of these important AS events. Moreover, the relationships between splice factors and these vital AS events were also analyzed to uncover possible pathways to influence the RFS of TNBC. In addition, we also studied the effectiveness of these AS events in overall survival (OS) prognosis for TNBC patients from all the aspects described above.

## Materials and Methods

### Data Collection

There are 150 TNBC patients who have records of RFS time of at least 30 days to be involved in this study. Their clinical information and gene expression data were collected from The Cancer Genome Atlas (TCGA) database^1^, including the information on OS time for a supplementary analysis. Additionally, their AS data were retrieved from TCGASpliceSeq (Ryan et al., 2016) database^2^.

### Identification and Functional Analysis of RFS-Related AS Events

Two procedures were conducted to identify RFS-related AS events in TNBC. First, the values of percent spliced in (PSI) across all the samples were checked to filter out uninformative AS events with mean PSI < 0.05 and standard deviation of PSI < 0.01. Second, univariate Cox regression analysis (Li et al., 2019) was applied to calculate the associations of each AS event with RFS. The significant RFS-related AS events were selected according to the criterion of *P* < 0.05. For the genes with these RFS-related AS events, Metascape (Zhou et al., 2019) software was used to annotate their potential biological functions and the involved pathways.

### Generation and Evaluation of AS Risk Factor

The least absolute shrinkage and selection operator (LASSO) method (Tibshirani, 1996) was used to select important RFS-related AS events and then to constitute AS risk factors for RFS prognosis across events in each AS type and all the AS events. Then, KM curves were used to evaluate the differences of RFS probabilities between the groups classified by the proposed AS risk factors, and the ROC curves were called to assess the prognosis performance of these AS risk factors. Then, the most effective AS risk factor was selected as a biomarker for RFS prognosis. Furthermore, the effectiveness of this AS risk factor in each clinical subgroup was tested to determine whether it contained independent and valuable information in RFS prognosis beyond clinical factors, such as tumor–node–metastasis (TNM) stages indicating the spread extent of tumor. If it did show important information, its combination with the clinical factors was then evaluated by ROC curves.

### Correlation Analysis Between Splicing Factors and Key AS Events

Overlapping with TNBC gene expression data, 494 SFs were collected from SpliceAid2 database (Piva et al., 2012) and three previous publications (Seiler et al., 2018; Wang et al., 2019; Gong et al., 2020), as shown in Supplementary Table S1. Then, we used univariate Cox regression model to identify RFS-related SFs (*P* < 0.05). Furthermore, we performed Pearson correlation analysis to find the significant associations between RFS-related SFs and AS events with the criteria of *P* < 0.05 and |*R*| > 0.300, which were visualized by Cytoscape (Otasek et al., 2019).

### The Performance of These RFS-Related AS Events in Overall Survival Prognosis

We performed an association analysis between RFS and OS in this part. Their strong correlations (*P* < 0.001 and |*R*| >0.6) reveal that it is promising to apply the RFS-related AS events in the OS prognosis field. Thus, we analyzed the effectiveness of the RFS-related AS events in OS prognosis from all the aspects described above. First, their relationships with OS of TNBC patients were analyzed by univariate Cox regression model. Then, these RFS-related AS events directly constituted another risk factor for OS prognosis, which was evaluated by KM and ROC curves across all the data and all kinds of clinical subgroups. Lastly, their regulations by SFs to influence the OS probability were also studied. All the statistical analyses involved in this study were completed in R (version 3.6.2).

## Results

### Overview of Samples and Informative AS Events

The demographic and histological data of TNBC cohorts involved in this study showed that 37.73% of patients underwent lymph node metastasis, 19.33% of patients experienced TNBC recurrence, and 26 deaths (17.33%) were confirmed during the period (Supplementary Table S2). Then, all the clinical data were tested by univariate Cox regression model to evaluate their prognostic roles. The results showed that TNM stages had significant correlations with the survival of TNBC which were then used in the following analysis. For the AS events, we first filtered out uninformative AS events and then identified a total of 45,421 informative AS events in 21,232 genes. The distributions of these informative AS events across different AS types (Supplementary Figure S1) revealed “exon skip” to be the most common AS type, which is consistent with previous studies (Florea et al., 2013; Kim P. et al., 2020).

### The Genes With RFS-Related AS Events Were Enriched in Splicing and TNBC-Related Pathways

**FIGURE 1:**
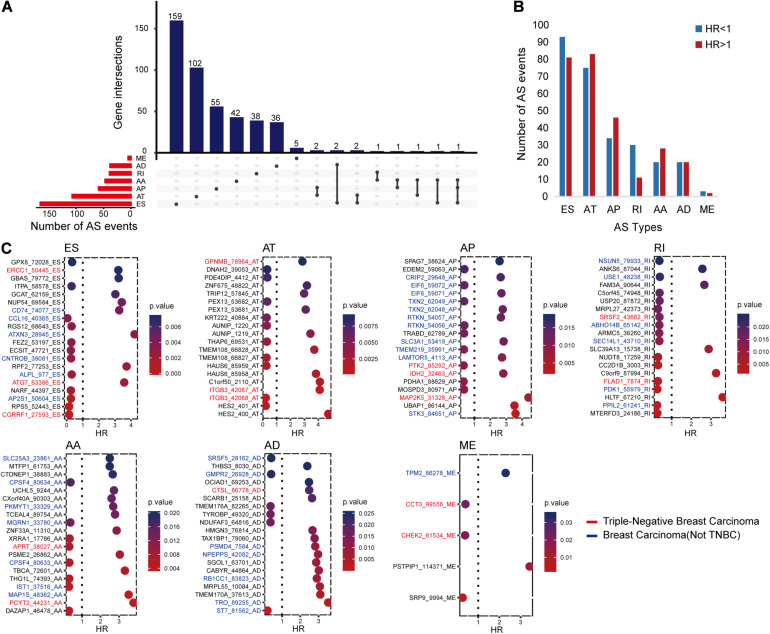
The distributions of recurrence-free survival (RFS)-related alternative splicing (AS) events. **(A)** Upset figure shows the number of AS events in exon skip (ES), alternate acceptor site (AA), retained intron, alternate terminator (AT), alternate donor site (AD), alternate promoter (AP), and mutually exclusive exons, respectively, and the distributions of these AS events in different genes. For example, there are 179 RFS-related ES events. Of all the genes with these RFS-related ES events, 159 genes only had ES events, two genes had ES and AD events, another two genes had ES and AT events, one gene had ES and AA events, and the last gene had ES, AA, and AP events simultaneously. **(B)** Bar chart displaying the favorable (HR, hazard ratio < 1) and unfavorable (HR > 1) events in each AS type. **(C)** Bubble charts presenting the top AS events in each type ranked by *P*-values. The AS events written in blue, red, and black are located in breast cancer-related genes, triple-negative breast cancer-related genes, and other genes, respectively. The relationships between these genes and diseases were collected from DisGeNET and some previous studies.

The univariate Cox regression analysis identified 546 RFS-related AS events in 448 genes, as shown in Figure 1A. All these AS events presented either an unfavorable consequence [hazard ratio (HR) > 1] or a favorable effect (HR < 1) to RFS time (Figure 1B).

In order to further understand the possible roles of these AS events in BC and even TNBC, we identified BC- or TNBC-associated genes among the 448 genes. The gene–disease relationships were downloaded from DisGeNET (2020.5^3^) (Piñero et al., 2020) or collected from other publications (Chen et al., 2014; Sesé et al., 2017; Yang et al., 2017; Lee et al., 2019; Osawa et al., 2019). It came out that there were 196 AS events in 166 BC-related genes. Among them, 48 AS events might have more chance to engage in TNBC occurrence and progression since they occurred in the 41 TNBC-associated genes. Figure 1C displays the top RFS-related AS events in each AS type ranked on the basis of *P*-values and also shows the information of their genes related with BC or TNBC.

**FIGURE 2:**
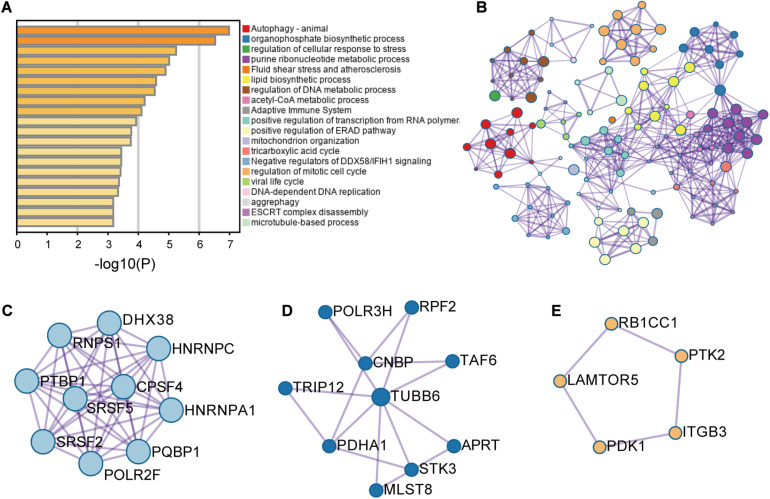
The enrichment analysis results of the genes with recurrence-free-survival-related alternative splicing events. **(A)** Enrichment analysis result. **(B)** Network analysis result. **(C–E)** Three modules were involved in the pathways of mRNA splicing, adaptive immune system and regulation of cellular response to stress, and autophagy, respectively.

These RFS-related genes were enriched in splicing-related pathways, BC-related or TNBC-related functions and others (Figures 2A,B). For example, Figure 2C displays the genes involved in mRNA splicing including different kinds of SFs such as SR protein family. Figure 2D presents the genes participating in the regulation of cellular response to stress and adaptive immune system, abnormity in which might cause BC (Varn et al., 2017; Yuan et al., 2017). Figure 2E exhibits the genes engaging in autophagy pathway. These genes are all BC-related genes based on DisGeNET, and autophagy has been reported to promote TNBC metastasis (Chen et al., 2019). All these analyses revealed the potential of these AS events to be involved in BC and even TNBC.

### Seven AS Events Have Significant Relationships With TNBC Survival

**FIGURE 3:**
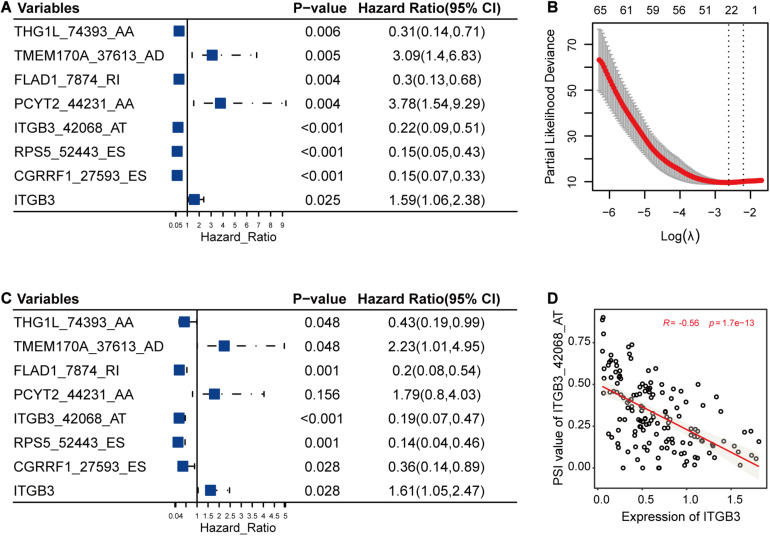
The selection and analysis of recurrence-free survival (RFS)-related alternative splicing (AS) events. **(A)** The associations of the seven AS events and one gene with RFS. **(B)** The seven AS events were selected by Lasso method. This panel shows the changes of partial likelihood deviance with *λ* values. The seven AS events were selected according to the most regularized model such that error is within one standard error of the minimum. **(C)** The associations of the seven selected AS events and one gene with overall survival. **(D)** One example for the correlations between AS events and their host genes. It revealed the possible relationships among the low PSI values of ITGB3_42068_AT, high expression levels of ITGB3, and high risk of triple-negative breast cancer survival.

In this part, LASSO Cox method was used to further reduce the size of RFS-related AS events and select more reliable ones across all the AS events. After the selection process, seven AS events (Figures 3A,B) were grouped into the final subset for RFS analysis. In addition, most of these RFS-related AS events were also significantly associated with OS of TNBC (*P* < 0.05) (Figure 3C).

Among the genes carrying the seven RFS-related AS events, CGRRF1 suppresses the growth of BC cells including TNBC cells (Lee et al., 2019), FLAD1 upregulates the HIF pathway which is associated with poor prognosis of TNBC (Chen et al., 2014; Yang et al., 2017), ITGB3 is important for tumor cell migration and TNBC survival (Sesé et al., 2017), and PCYT2 activity has been shown to be increased in BC cells, enabling them to adapt to metabolic stress (Zhu and Bakovic, 2012; Mota et al., 2015). Moreover, the differential analysis results were also consistent with the above-mentioned descriptions, showing that the expression levels of CGRRF1 (2.80e-7), FLAD1 (3.70e-7), ITGB3 (9.40e-3), and PCYT2 (0.02) were significantly varied in TNBC cohorts compared to healthy controls. From previous literature and the analysis results, we could notice the pivotal functions of these genes in TNBC. Accordingly, the abnormal AS events in these crucial genes might alter their original functions and then have an effect on the survival of TNBC. Furthermore, we calculated the correlations between the PSI values of these AS events and the expression levels of their host genes. As shown in Figure 3D, the low PSI values of ITGB3_42068_AT were significantly associated with the high expression of ITGB3, both of which might lead to a poor prognosis of TNBC survival (Figure 3).

### The AS Risk Factors Showed Strong Prognosis Ability for TNBC Survival

**FIGURE 4:**
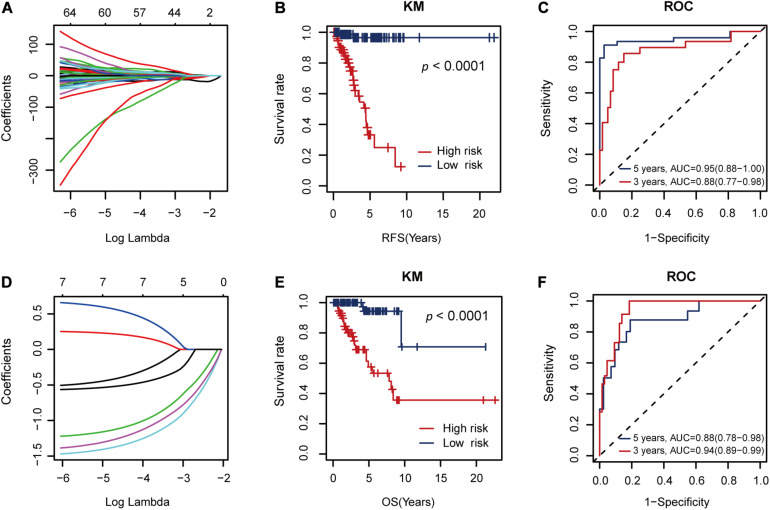
The performance of the proposed alternative splicing (AS) risk factors. **(A)** The coefficients from the Lasso fit represent the contributions of different AS events to recurrence-free survival (RFS) prognosis. Then, these AS events constituted an AS risk factor for RFS prognosis under the optimal *λ* value (log*λ* = –2.20, Figure 3B) using the formula (1) defined in the Supplementary Material. **(B)** Kaplan–Meier (KM) curves for the low and high RFS risk groups classified by the AS risk factor. **(C)** Receiver operating characteristic (ROC) curves showing the RFS prognosis ability of the AS risk factor. **(D)** The coefficients from the Lasso fit represent the contributions of the seven AS events to overall survival (OS) prognosis. Then, these AS events constituted another AS risk factor for OS prognosis under the optimal *λ* value (log*λ* = –3.25) using the formula (2) defined in the Supplementary Material. **(E)** KM curves for the low and high OS risk groups classified by the AS risk factor. **(F)** ROC curves showing the OS prognosis ability of the AS risk factor.

The seven RFS-related AS events constituted a risk factor using formula 1 in the Supplementary Material (Figure 4A). This risk factor could classify the low and high RFS risk groups significantly (*P* < 0.001), as shown in Figure 4B. Moreover, it also achieved areas under the ROC curve (AUCs) of 0.88 (0.77–0.98) and 0.95 (0.88–1.00) for 3- and 5-year RFS prediction, respectively, as shown in Figure 4C. Since this AS risk factor exhibited strong prognosis ability in RFS prediction than the other seven AS risk factors obtained from only one AS type as shown in Supplementary Figures S2, S3, it was proposed as the final biomarker for RFS prognosis of TNBC.

In addition, these seven RFS-related AS events could also constitute another AS factor using formula 2 in the Supplementary Material for OS prognosis (Figure 4D). Similarly, it showed strong discriminative ability between the low and high OS risk groups (Figure 4E) and relatively high AUC values of 0.94 (0.89–0.99) and 0.88 (0.78–0.98) for 3- and 5-year OS prediction, respectively (Figure 4F).

**FIGURE 5:**
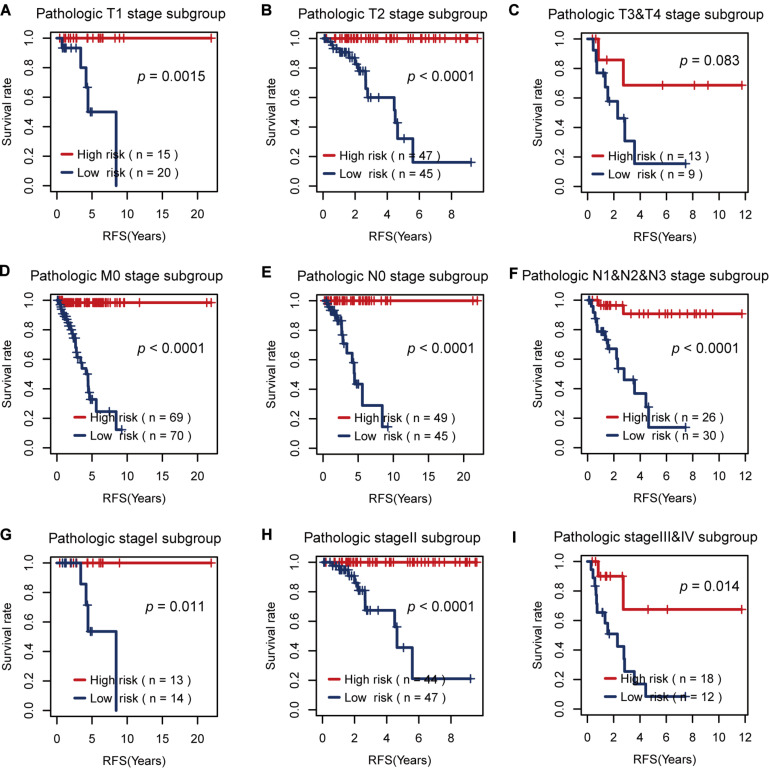
The usefulness of the alternative splicing (AS) risk factor for recurrence-free survival (RFS) prognosis in different categories grouped by clinical tumor–node–metastasis stages. **(A–I)** Kaplan–Meier curves of the AS risk factor in the groups of T1, T2, T3 + T4, M0, N0, N1 + N2 + N3, stage I, stage II, and stage III + stage IV. It showed that this risk factor still had the power to significantly differentiate between the low and high RFS risk groups even under similar clinical conditions.

**FIGURE 6:**
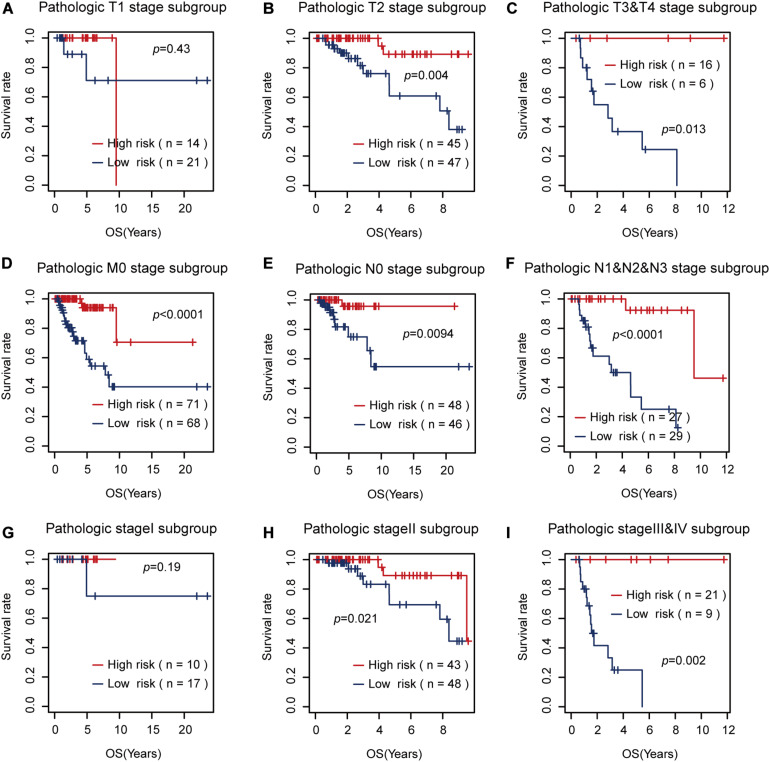
The usefulness of the alternative splicing (AS) risk factor for overall survival (OS) prognosis in different categories grouped by clinical tumor–node–metastasis stages. **(A–I)** Kaplan–Meier curves of the AS risk factor in the groups of T1, T2, T3 + T4, M0, N0, N1 + N2 + N3, stage I, stage II, and stage III + stage IV. It showed that this risk factor still had the power to significantly differentiate between the low and high OS risk groups in most of the clinical sub-groups.

Then, we tested the effects of these two risk factors in different categories grouped by clinical factors, namely, the T, M, N, and pathological stages. The risk factor for RFS survival prognosis showed strong discriminative ability in every kind of clinical subgroup (*P* < 0.05), as shown in Figure 5. Additionally, even though the risk factor for OS prognosis could not significantly differentiate between the high and low OS risk groups for the patients with T1 or pathological I, it did show a good performance in other clinical subgroups with more severe tumor conditions (Figure 6). Thus, the results in the two figures indicated that these AS risk factors carried valuable and independent information in the fields of TNBC survival prognosis beyond the clinical TNM stages.

**FIGURE 7:**
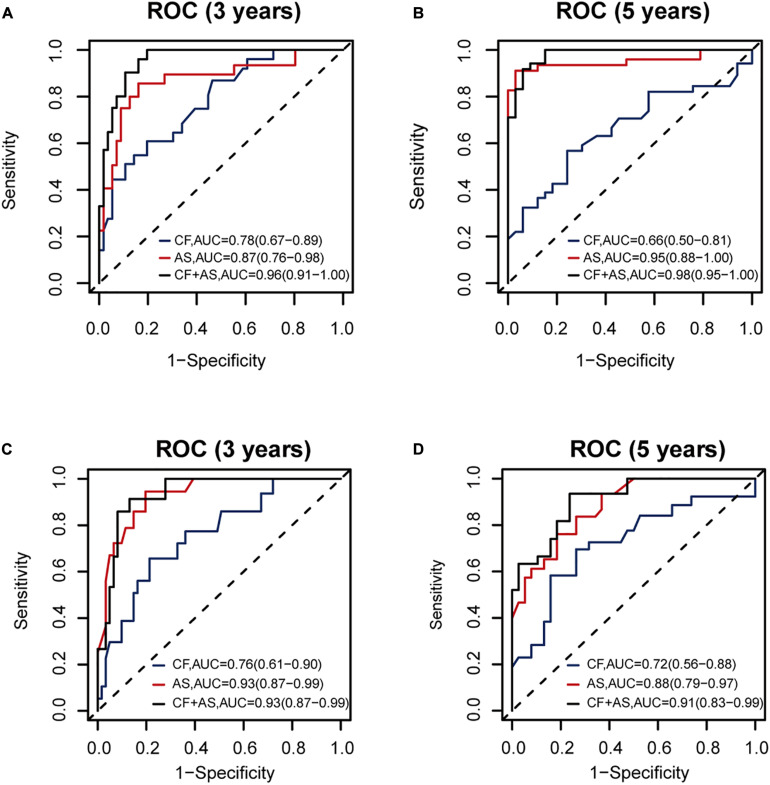
The performance of the proposed alternative splicing (AS) risk factors combined with clinical tumor–node–metastasis (TNM) stages. The performance of clinical TNM stages, AS risk factors, and the combined features are shown **(A)** for 3-year recurrence-free survival (RFS), **(B)** for 5-year RFS, **(C)** for 3-year overall survival (OS), and **(D)** for 5-year OS prognosis, respectively, based on 140 samples with complete clinical information.

Thus, we combined the AS risk factors and TNM stages for RFS and OS prognosis. The combined features had better prognosis ability than alone, realizing AUC values of 0.96 (0.91–1.00), 0.98 (0.95–1.00), 0.93 (0.87–0.99), and 0.91 (0.83-0.99) for 3-year RFS, 5-year RFS, 3-year OS, and 5-year OS prediction, respectively (Figure 7).

All the above-mentioned analyses underlined the important roles of the seven AS events in the survival prognosis of TNBC patients. Then, we continued to study the potential regulations of these AS events by SFs in the following part to discover the possible regulation pathways influencing the survival of TNBC.

### Regulation of AS Events by Splicing Factors to Influence the Survival of TNBC

**FIGURE 8:**
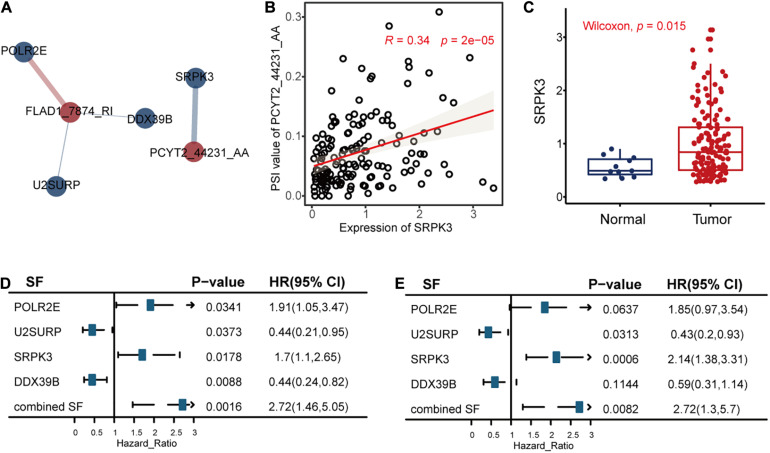
The correlation analysis results between the alternative splicing (AS) events and recurrence-free survival (RFS)-related splicing factors (SFs). **(A)** The significant (*P* < 0.05 and | *R*| > 0.3) positive associations (blue line) and negative associations (red line) between AS events (red circle) and RFS-related SFs (blue circle). The width of the lines represented the correlation coefficients between AS events and RFS-related SFs. **(B)** The relationships between the expression levels of SRPK3 and the percent spliced in values of PCYT2_44231_AA. Other associations are displayed in Supplementary Figure S4. **(C)** The differential expression analysis of SRPK3 between tumor samples and healthy controls. **(D,E)** The correlation results between the SFs and RFS or OS of triple-negative breast cancer, respectively.

The univariate Cox regression model identified 32 RFS-related SFs. After that, we calculated the correlations between the seven AS events and 32 SFs to reveal the possible regulations of these splicing events by SFs, as shown in Figure 8, Supplementary Figure S4, and Supplementary Table S3.

We took the significant association between PCYT2_44231_AA and SF SRPK3 as an example. The analysis results revealed that SRPK3 may upregulate the splicing of PCYT2_44231_AA (Figure 8B) and then lead to poor TNBC survival. This hypothesis is in consistency with the function of SRPK3 reported in a previous study that SRPK3 was experimentally confirmed by growth inhibition following its knockdown by siRNAs in the TNBC cell line of CAL51 (Szwajda et al., 2015), accordant with the differential expression analysis result in Figure 8C that SRPK3 was upregulated in TNBC patients compared to healthy controls, in line with the univariate Cox regression analysis results of SRPK3 that the high expression levels of this gene were related to high TNBC survival risk (Figures 8D,E), and in agreement with the survival analysis result of PCYT2_44231_AA in Figure 3A that the high PSI values of PCYT2_44231_AA were associated with a high risk of RFS. Even though the PSI values of PCYT2_44231_AA were not significantly correlated with OS as analyzed by the Cox regression method (Figure 3C), its contributions to OS prognosis by LASSO method (formula 2 in the Supplementary Material) still revealed the positive relationships between PCYT2_44231_AA and OS. Then, we might connect the PCYT2_44231_AA regulated by SRPK3 to the TNBC survival.

Additionally, there are three other significant associations (Supplementary Figures S4B–D) showing the possible regulations of POLR2E, U2SURP, and DOX39B on FLAD1_7874_RI. We defined two factors integrating the co-effects of the three SFs on TNBC survival according to formula 10 and 11 in the Supplementary Material. We hypothesized that the three SFs may work together to downregulate the splicing of FLAD1_7874_RI to have an effect on the survival of TNBC (Supplementary Figure S4). This hypothesis is consistent with the involvement of POLR2E and U2SURP in BC discovered in previous studies (Brough et al., 2018; An et al., 2020), in agreement with the survival analysis results of the combined factors (Figures 8D,E) that their high values were significantly correlated with the high risk of TNBC survival, and also accordant with the Cox analysis results that the low PSI values of FLAD1_7874_RI were related to the high risk of TNBC survival (Figures 3A,C). All these might present the promising roles of these AS events regulated by SFs to influence TNBC survival.

## Discussion

The survival analysis of TNBC has gotten enough attention from researchers around the world due to its severity and poor survival outcome. There are several studies focusing on the roles of clinical and treatment factors (Xiao, 2016; Kim et al., 2019; Wen et al., 2020), radiomics features (Kim S. et al., 2020; Koh et al., 2020), circulating tumor cells (Karhade, 2012), and gene expressions (Kim et al., 2016; Purrington et al., 2020; Wu et al., 2020) in the survival prognosis of TNBC. The research groups of these studies tried to find the significant risk factors for TNBC survival and further contributed to the treatment of TNBC. Since TNBC is a complex disease which lacks targeted therapies, shows high recurrence ratio, and displays low survival probability, a novel risk factor focusing on genetic variations is needed to reveal the potential molecular mechanisms related to TNBC survival. Since previous studies (El Marabti and Younis, 2018; Zhang et al., 2019; Bonnal et al., 2020; Gong et al., 2020) showed the promising roles of the aberrant AS events in cancer, we tried to explore the functional impacts of AS in the survival of TNBC.

Furthermore, one recent study (Gong et al., 2020) deeply analyzed the influence of AS events on the OS of TNBC. Compared to its best predictor model with AUC value of 0.949 for 3-year OS prediction, our AS risk factor combining the seven RFS-related AS events showed a similarly high result with an AUC value of 0.94 for 3-year OS prognosis (Figure 3F). Additionally, our proposed model integrating the AS risk factor and clinical information could predict 3- and 5-year RFS probability precisely with AUC values of 0.96 and 0.98, respectively, as well.

In detail, our AS risk factors contained seven informative AS events, which were CGRRF1_27593_ES, RPS5_52443_ES, ITGB3_42068_AT, FLAD1_7874_RI, PCYT2_44231_AA, TMEM170A_ 37613_AD, and THG1L_74393_AA. They showed remarkable prognosis ability alone and better performance in survival prognosis when combined with clinical factors. Among the genes with these AS events, four genes (Zhu and Bakovic, 2012; Chen et al., 2014; Mota et al., 2015; Sesé et al., 2017; Yang et al., 2017; Lee et al., 2019) have been reported to be related with TNBC. Moreover, we compared the expressions of these genes between 198 TNBC patients and 67 non-TNBC BC patients (GSE76275). The abnormal expressions of TMEM170A, FLAD1, ITGB3, and CGRRF1 (Supplementary Figure S5) may reveal their relationships with poor survival since TNBC shows worse survival prognosis than other types of BCs. Next, we also compared the gene expressions between 65 TNBC samples and 33 paired healthy breast tissues (GSE76250). It showed that FLAD1, PCYT2, CGRRF1, and RPS5 were dysregulated in TNBC tumors (Supplementary Figure S5). Thus, the key functions of these genes related to TNBC showed the high participation opportunities of these AS events in TNBC.

Furthermore, we performed a correlation analysis between the AS events and their host genes. Then, we discovered significantly negative relationships between ITGB3_42068_AT splicing and ITGB3 expression, which were all remarkably related to TNBC survival. It revealed that this splicing event might cause the differential expressions of this gene and then affect cell migration and survival in TNBC. Besides this, we also conducted a correlation analysis between the AS events and their related isoforms. We discovered the negative associations between PSI values of TMEM170A_37613_AD and the expressions of ENST00000568559.1 (uc002fed.1) which were weakly related to the survival of TNBC (Supplementary Figure S6). Since transmembrane protein TMEM170A is a regulator of endoplasmic reticulum and nuclear envelope morphogenesis in human cells (Christodoulou et al., 2016) which has associations with TNBC-related autophagy (Bernales et al., 2006), the AS in TMEM170A may affect its isoform expressions to influence TNBC survival.

The abnormally higher expressions of SRPK3 in TNBC samples compared to healthy controls shown in the two datasets of TCGA (Figure 8C) and GSE76250 (Supplementary Figure S5) may reveal the possibility of the SFs to be involved in TNBC survival. Then, we performed a correlation analysis between the PSI values of these splicing events and the expression levels of SFs. It indicated that SFs might abnormally regulate AS events, leading to high invasiveness, fast tumor growth, and low survival of TNBC (Park et al., 2019), such as the regulation of PCYT2_44231_AA by SRPK3 and the co-effects of POLR2E, U2SURP, and DOX39B on FLAD1_7874_RI to influence TNBC survival as concluded in this work. Overall, AS events regulated by SFs might act as a kind of survival biomarker for TNBC, which could be helpful to classify the high and low survival risk of TNBC patients to provide precise medicine.

## Data Availability Statement

The original contributions presented in the study are included in the article/Supplementary Material. Further inquiries can be directed to the corresponding author/s.

## Ethics Statement

Ethical review and approval was not required for the study on human participants in accordance with the local legislation and institutional requirements. Written informed consent for participation was not required for this study in accordance with the national legislation and the institutional requirements.

## Author Contributions

SW, JW, JC, XW, and XZhou contributed to the conception and study design. JW contributed to the acquisition of data. SW, JW and XZhu contributed to the analysis and interpretation of data. SW contributed to the drafting of the manuscript. SW, JW, XZhu, JC, XZhou, XW, and LH contributed to the critical revision of the manuscript. JW and XZhu contributed to the statistical analysis. LH supervised the study. All authors contributed to the article and approved the submitted version.

## Conflict of Interest

The authors declare that the research was conducted in the absence of any commercial or financial relationships that could be construed as a potential conflict of interest.

## Footnotes

1 https://tcga-data.nci.nih.gov/tcga/

2 https://bioinformatics.mdanderson.org/TCGASpliceSeq/

3 https://www.disgenet.org/static/disgenet_ap1/files/downloads/all_gene_disease_associations.tsv.gz
