# Supplementary material for: The Functional Impact of Alternative Splicing on the Survival Prognosis of Triple-Negative Breast Cancer
Source: Front Genet. 2021 Jan 14;11:604262. doi: 10.3389/fgene.2020.604262 (PMC7841428; doi:10.3389/fgene.2020.604262)
Supplement: Supplementary file 2 [file Data_Sheet_1.docx]

Supplementary Material

# AS risk factors

AS risk factor used for RFS prognosis of TNBC

$$\begin{matrix} Riskscore-combined \\ =-18.1050\times CGRRF1\_27593\_ES-0.0261\times RPS5\_52443\_ES \\ -0.4516\times ITGB3\_42068\_AT+1.3300\times PCYT2\_44231\_AA \\ -0.1603\times FLAD1\_7874\_RI+0.8730\times TMEM170A\_37613\_AD \\ -4.3642\times THG1L\_74393\_AA \end{matrix}$$

AS risk factor for OS prognosis of TNBC

$$\begin{matrix} Riskscore-combined \\ \text{=}-0.5394\times THG1L\_THG1L\_74393\_AA+0.2629\times TMEM170A\_37613\_AD \\ -1.2570\times FLAD1\_7874\_RI+0.6929\times PCYT2\_44231\_AA \\ -1.5078\times ITGB3\_42068\_AT-1.4379RPS5\_52443\_ES \\ -0.5809\times CGRRF1\_27593\_ES \end{matrix}$$

Other seven AS risk factors calculated based on only one type of AS events for RFS prognosis of TNBC.

$$\begin{matrix} Riskscore-ES \\ =-0.6898\times RPS5\_52443\_ES+0.4201\times ZNF253\_48693\_ES \\ -3.6954\times H2AFV\_79576\_ES-0.2552\times SULT1A1\_35821\_ES \\ -24.1543\times CGRRF1\_27593\_ES \end{matrix}$$

$$\begin{matrix} Riskscore-AT \\ =-1.3527\times ITGB3\_42068\_AT+1.2734\times C1orf50\_2110\_AT \\ +0.4465\times HES2\_400\_AT-0.2407\times ZNF506\_48680\_AT \\ -0.1034\times THAP6\_69531\_AT-0.9137\times RABL5\_81057\_AT \\ +0.0003\times RABL5\_81059\_AT \end{matrix}$$

$$\begin{matrix} Riskscore-AP \\ =3.8221\times NUP50\_62637\_AP+0.2974\times STK3\_84651\_AP \\ -2.073\times PTK2\_85292\_AP-0.2434\times RARG\_21979\_AP \\ +2.3903\times PBX1\_8780\_AP+0.4911\times EIF6\_59071\_AP \\ -0.0012\times EIF6\_59072\_AP+9.8081\times LAMTOR5\_4113\_AP \\ +9.8096\times MDH1\_53732\_AP+0.1012\times SAYSD1\_76036\_AP \end{matrix}$$

$$\begin{matrix} Riskscore-RI \\ =1.8352\times HLTF\_67210\_RI-1.8691\times FLAD1\_7874\_RI \end{matrix}$$

$$\begin{matrix} Riskscore-AA \\ =2.9068\times PCYT2\_44231\_AA-0.9836\times CPSF4\_80633\_AA \\ +0.4363\times CTDNEP1\_38883\_AA-10.5049\times THG1L\_74393\_AA \end{matrix}$$

$$\begin{matrix} Riskscore-AD \\ =1.7700\times TMEM170A\_37613\_AD-0.4427\times UBL7\_31722\_AD \\ \text{ }+2.3044\times PSMD4\_7584\_AD+0.4049\times TRO\_89255\_AD \end{matrix}$$

$$\begin{matrix} Riskscore-ME \\ =-9.4330\times SRP9\_9994\_ME-0.8007\times CHEK2\_61534\_ME \\ +1.8010\times TPM2\_86278\_ME-1.2337\times CCT3\_99558\_ME \\ +6.2608\times PSTPIP1\_114371\_ME \end{matrix}$$

# The co-effects of SFs to TNBC survival

The co-effects of SFs to influence the RFS survival of TNBC

$$Riskscore\text{ }=\text{ }0.4133\times POLR2E-0.3677\times U2SURP-0.5679\times DDX39B$$

The co-effects of SFs to influence the OS survival of TNBC

$$Riskscore\text{ }=\text{ }0.4012\times POLR2E-0.5678\times U2SURP-0.1912\times DDX39B$$

# Supplementary Figures

## Supplementary Figure 1


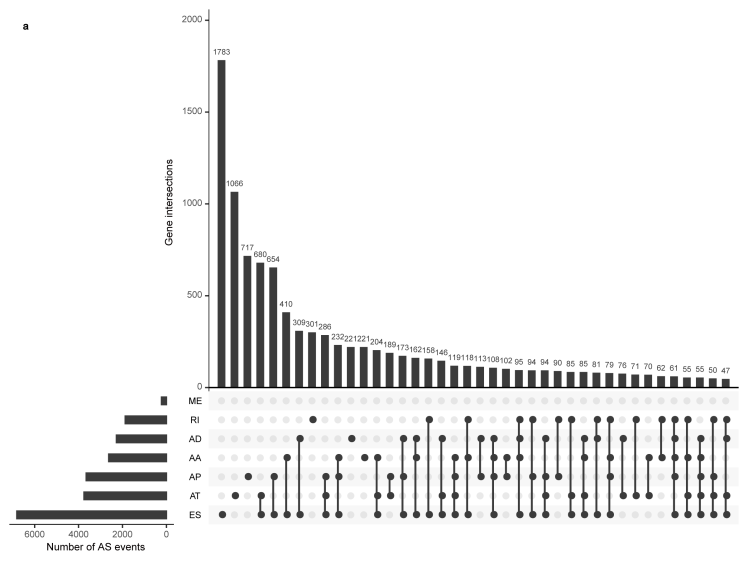


**Supplementary Figure 1.** The distributions of the informative AS events in the seven AS types. There are a total of 45,421 AS events in 21,232 genes in patients with TNBC, including 17,702 ES (exon skip) events in 6,812 genes, 3,731 AA (alternate acceptor site) events in 2,628 genes, 2,802 RI (retained intron) events in 1,878 genes, 8,595 AT (alternate terminator) events in 3,755 genes, 3,246 AD (alternate donor site) events in 2,278 genes, 9,112 AP (alternate promoter) events in 3,654 genes and 233 ME (mutually exclusive exons) events in 227 genes.

## Supplementary Figure 2


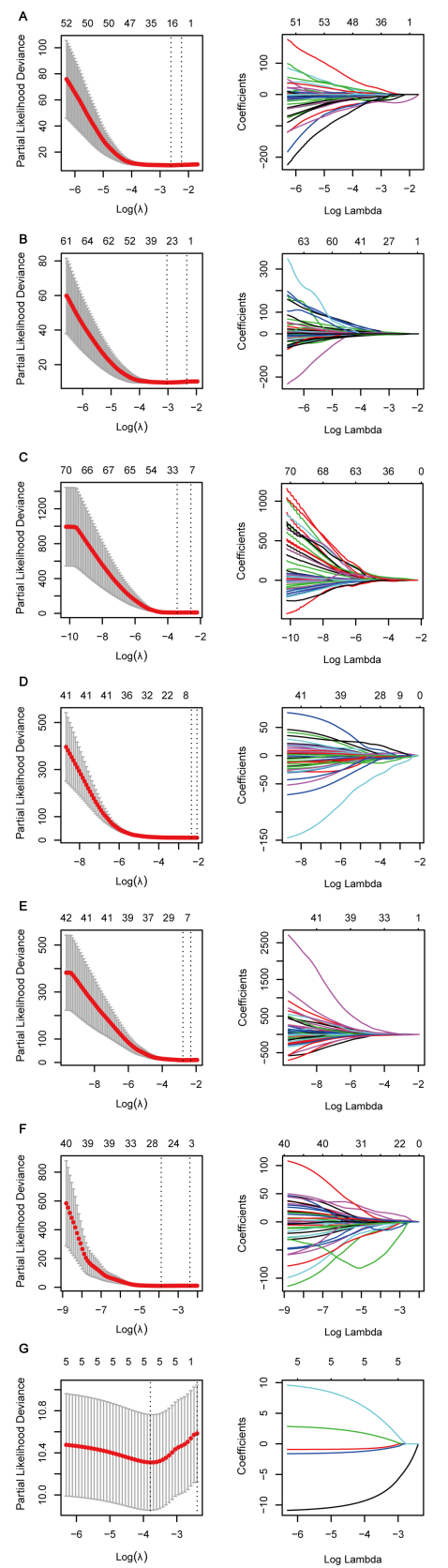


**Supplementary Figure 2.** The lasso selection processes for ES (A), AT (B), AP (C), RI (D), AA (E), AD (F) and ME (G) events respectively.

## Supplementary Figure 3


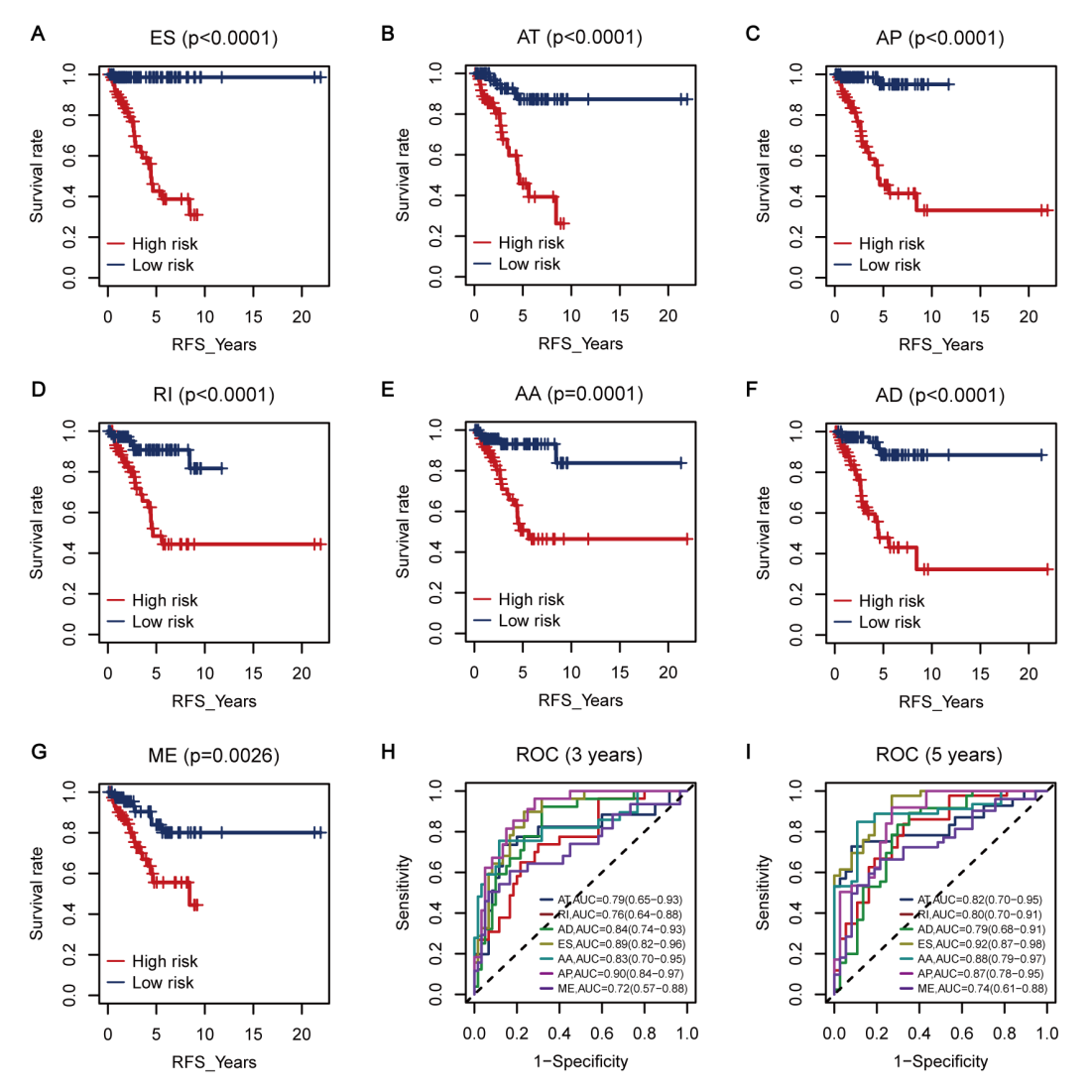


**Supplementary Figure 3.** The performance of AS risk factors formed on the basis of only ES, AT, AP, RI, AA, AD, and ME events. (A-G) KM curves for the seven risk factors. (H-I) ROC results of the seven risk factors for 3- and 5-year RFS risk prediction respectively.

## Supplementary Figure 4


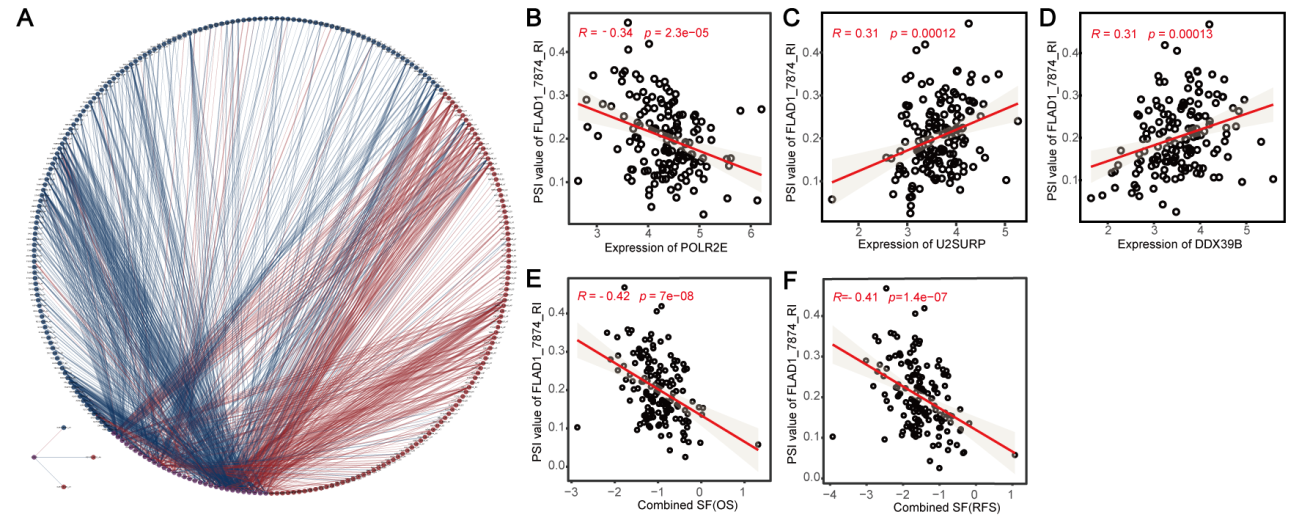


**Supplementary Figure 4.** The correlation analysis results between AS events and RFS-related splicing factors. (A) This panel shows the significant (P<0.05 and |R|>0.3) positive relationships (blue line) and negative relationships (red lines) between 138 favorable (blue circle) or 102 unfavorable (red circle) RFS-related AS events and 32 RFS-related SFs (purple circle). The width of lines represented the correlation coefficients between AS events and SFs. (B-D) The significant associations between the expression levels of three SFs and the PSI values of FLAD1_7874_RI. (E-F) The relationships between the two combined factors and FLAD1_7874_RI for RFS and OS prognosis respectively. The two combined SFs were defined according to Formula 10 and 11 in the Supplementary File.

## Supplementary Figure 5


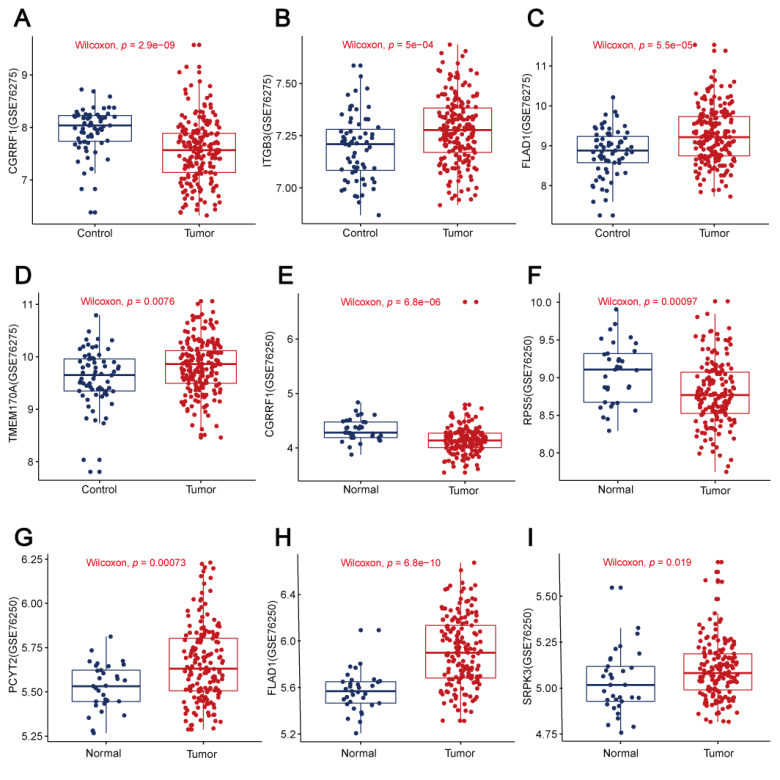


**Supplementary Figure 5.** The differential expressed genes in TNBC samples compared to others on the basis of external datasets (GSE76275 and GSE76250). (A-D) CGRRF1, ITGB3, FLAD1 and TMEM170A were differential expressed in TNBC patients compared to non-TNBC breast cancer patients in the dataset of GSE76275. (E-I) CGRRF1, RPS5, PCYT2, FLAD1 and SRPK3 were dys-regulated in TNBC samples compared to healthy breast tissues.

## Supplementary Figure 6


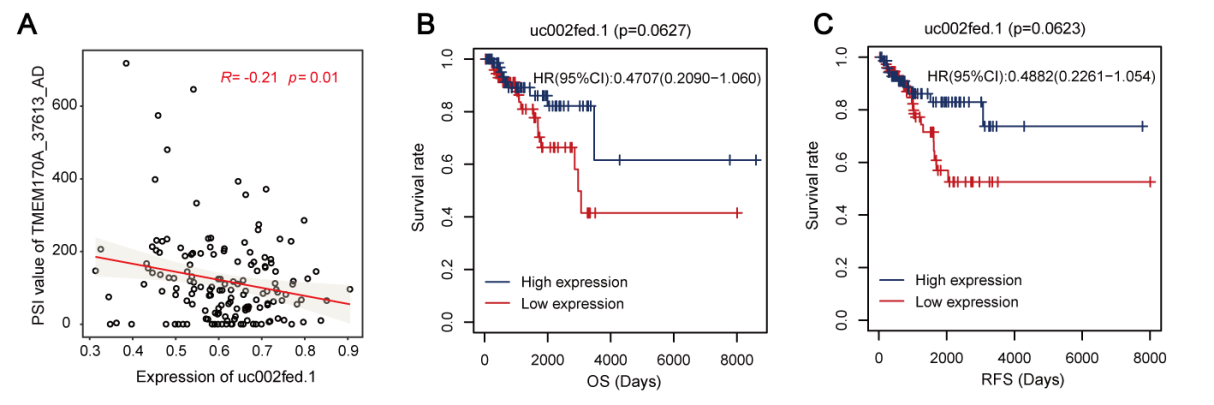


**Supplementary Figure 6.** The relationships between AS events, isoform expressions and TNBC survival. (A) The negative correlations between the PSI values of TMEM170A_37613_AD and the expressions of ENST00000568559.1 (uc002fed.1). (B-C) The expressions of uc002fed.1 showed weak associations with the OS and RFS of TNBC.
